# Incidence of common respiratory pathogens among patients with severe acute respiratory infection during COVID-19 pandemic in Egypt

**DOI:** 10.1038/s41598-025-98907-y

**Published:** 2025-05-05

**Authors:** Nermeen Talaat Fahmy, Mohamed Abdel-Salam Elgohary, Wael A. Hassan, Ahmed Abd El-Raouf, Sara H. A. Agwa, Hala Hafez, Amr Yusuf Ali, Fadya M. El-Garhy, Omnia M. Abdel-Haseb, Tokka M. Hassan, Yasmeen K. Farouk, Hoda Ez, Ihab Shehad Habil, Ossama Ibrahim Mansour, Ashraf Omar Mahmoud, Mahmoud El Meteini, Samia Abdou Girgis, Khaled Amer

**Affiliations:** 1 Egypt Center for Research and Regenerative Medicine (ECRRM), Cairo, Egypt; 2 Medical Ain Shams Research Institute, Ain Shams University, Cairo, Egypt; 3 Faculty of Medicine, Ain Shams University, Cairo, Egypt; 4 Ministry of Health and Population, Cairo, Egypt; 5 Ain Shams University Hospital, Cairo, Egypt

**Keywords:** Respiratory pathogens, SARI, COVID-19, Pathogens, Virology

## Abstract

Severe Acute Respiratory Infection poses a significant threat to human health being a major cause of morbidity and mortality. The rate of co-infection among the underlying pathogens is unknown. During COVID-19 pandemic, reports for respiratory pathogens co-circulations in developing countries were limited. Identification of respiratory pathogens is paramount for effective patient management as early detection decreases the risk of mortality and morbidity. This is the first report to investigate the incidence of respiratory pathogens co-infection among patients with SARI during COVID-19 pandemic in Egypt. Clinically SARI patients were recruited from October 2020 to June 2022. Nasopharyngeal swabs were collected to detect SARS-CoV-2 followed by 33 respiratory pathogens identification using RT-PCR. Of 599 samples tested, 27% (158/599) patients were positive for COVID-19, in which 75.9% (120/158) patients were co-infected with other respiratory pathogens. In total, 31 pathogens were identified with a detection rate of 75% (450/599) among positive and negative COVID-19 patients. Bacterial co-infections rate was 39%, in which *Klebsiella pneumoniae* (5.3%) is the most common, while viral co-infections rate was 61%, in which Human Coronavirus HKU1 (6.2%) is the most common. Adenovirus, human rhinovirus and RSV were only detected in 70/11.7%, 50/8.3% and 41/6.8% of cases, respectively. Early detection and management of respiratory pathogens co-infection are crucial for effective patient management and preparedness for future pandemics.

## Introduction

In low- and middle-income countries, the burden of severe acute respiratory infections (SARI) is high, and associated with significant morbidity and mortality^1,2^. High attention has been paid to the etiological tracing of respiratory tract infection since the advent of COVID-19^3^.

The emerging of the highly pathogenic severe acute respiratory syndrome (SARS-CoV-2) in December 2019 has widely spread to become a major public health threat with a significant number of deaths worldwide^4^. During COVID-19 pandemic period, co-circulation of respiratory pathogens in developing countries were under-reported^5^. SARS-CoV-2 screening by real-time polymerase chain reaction (RT-PCR) has been an increased focus among patients with respiratory symptoms in these countries. Testing for other respiratory pathogens that cause SARI has been neglected. SARI clinical symptoms are almost similar to COVID-19 that can obstruct the diagnosis of patients with suspected COVID-19^6^. Detection of other respiratory pathogens is crucial to prevent adverse outcomes, and reduce antibiotic usage for better case management. Disparities in the prevalence of respiratory pathogens co-infection among countries have been reported in several studies, attributed to discrepancies in age groups, geographical location, seasons, and other factors^7,8^.

Several molecular methods have been described for the diagnosis of respiratory infections^9–11^. Multiplex Real time PCR (rRT-PCR) commercial panels were recently used for detecting a wide range of respiratory pathogens with high sensitivity, efficiency and time saving which made it the method of choice such as FilmArray^®^ Respiratory Panel (Biofire Diagnostics, Salt Lake City, UT), Respiratory Multi Well System MWS r-gene^®^ Range (BioMerieux) and Fast-Track Diagnostics FTD-33. Performance of these new multiplex panels were reported in few studies^9,10,12^.

Recent studies have shown the presence of respiratory pathogens co-infection during COVID-19 pandemic waves^13–15^. The most common bacterial co-infections reported in COVID-19 patients were *Chlamydophila pneumoniae*,* Mycoplasma pneumoniae*,* Streptococcus pneumoniae*, and S*taphylococcus aureus*^16^ and for viral co-infection; influenza A virus (IAV), influenza B virus (IBV), rhinovirus/enterovirus (EV), respiratory syncytial virus (RSV) and metapneumovirus were reported^17^. Hashemi et al.^18^ study has detected positive respiratory viruses in most of COVID-19 patients tested in which influenza A virus rate was 22.3%. Roh et al.^19^ reported 8.8% respiratory pathogens co-infection in which *Mycoplasma pneumoniae* and adenovirus were detected in COVID-19 patients. In a Chinese study, 5.8% co-infection was detected in COVID-19 patients, and 18.4% co-infection was detected in patients without COVID-19^15^.

To our knowledge, there was a lack in the studies that described the respiratory pathogen co-infection among SARI patients during COVID-19 pandemic in the region. Only one survey study focused on viral co-infection has been reported among children that was admitted with SARI and negative for COVID-19 in Egypt. This study showed 92.1% viral co-infection rate in which Rhinovirus was the main cause of death among children^20^.

Here, is the first report that aimed to evaluate the incidence of common respiratory pathogens in SARI patients in Egypt during COVID-19 pandemic, and identify groups at risk of severe disease.

## Materials and methods

### Patient and sample collection

The study was approved by the Research Ethics Committee at Ain Shams University Faculty of Medicine (IRB no.: FMASU P94a/2020–2021). All methods were performed in accordance with the relevant guidelines and regulations. Patients who met the WHO standard SARI case definition [Patients with acute respiratory infection who have history of fever (or measured fever of ≥ 38 °C), cough and onset of symptoms within the last 10 days and require hospitalization]^17^ were recruited from the internal medicine, chest department, pediatric hospital, geriatric hospital and intensive care units (ICUs) in Ain Shams University Hospitals, Cairo, Egypt during the period from October 2020 till June 2022. A questionnaire that covered history of fever and/or respiratory symptoms, travelling history, any underlying lung disease, history of chronic or immunocompromised conditions, and outcome was completed for each subject.

A total of 599 nasopharyngeal swab samples were collected from patients after signing informed consents. Samples selected in this study originated from patients with severe acute respiratory symptoms aged less than 15 to more than 60 years old of both genders. Collected samples were stored in 2 ml viral transport media (VTM) and transported to Egypt Center for Research and Regenerative Medicine (ECRRM) and Faculty of Medicine, Ain Shams University laboratories for molecular evaluation.

### Nucleic acid purification

Nucleic acid was extracted using Prepito NA Body Fluid Kit (Perkin Elmer, USA) following the manufacturer’s protocol with a final elution of 100 µl. Internal control (supplied with RT-PCR kit) was added to each sample. Extracted nucleic acid concentration and purity was measured by NanoDrop™ One Microvolume UV-Vis Spectrophotometer (ThermoFisher, USA).

### Real time polymerase chain reaction (RT-PCR)

Extracted samples were tested for SARS-CoV-2 using ProLab/Cer Test Biotech VIASURE SARS-CoV-2 (N1 + N2) Real Time PCR Detection kit (CerTestBiotec, S.L, Spain), targeting two conserved regions of nucleocapsid protein gene (N1 and N2) following manufacturer’s instructions. Samples were processed with appropriate negative, internal, and positive controls.

Fast Track Diagnostic (FTD^®^) 33 multiplex kit was used for detection of 33 respiratory pathogens using Real time PCR including influenza A virus (IAV), influenza B virus (IBV), influenza C virus (IAC), influenza A (H1N1) virus swine lineage (IAV (H1N1) swl), human parainfluenza viruses (HPIV) 1, 2, 3 and 4, human coronaviruses (HCOV) NL63, 229E, OC43 and HKU1, human metapneumoviruses A and B (HMPVA and B), human rhinovirus (HRV), respiratory syncytial viruses A and B (HRSVA and HRSVB), human adenovirus (HAdV), enterovirus (EV), human parechovirus (HPeV), human bocavirus (HBoV), *Pneumocystis jirovecii* (*P. Jirovecii*), *Mycoplasma pneumoniae* (*M. pneumoniae*), *Chlamydophila pneumoniae (C. pneumoniae)*,* Streptococcus pneumoniae* (*S. pneumoniae*), *Haemophilus influenzae type B (*H. Influenza B), *Staphylococcus aureus* (*S. aureus*), *Moraxella catarrhalis (M. catarrhalis)*,* Bordetella spp.*,* Klebsiella pneumoniae* (*K. pneumoniae*), *Legionella pneumophila/ Legionella longbeachae*,* Salmonella spp.* and *Haemophilus* influenza (*H. influenza*).

Eight multiplex RT-PCR reactions were prepared; each PCR reaction contained 10uL of nucleic acids template, 12.5µL of the buffer, 1.5µL of primer/probe mix, and 1µL of enzyme. Using an ABI 7500 Real Time PCR System (Applied Biosystems™, USA), reaction mixture was initially incubated at 50 °C for 15 min, 94 °C for 1 min, followed by 40 cycles of: 94 °C for 8 s, and 60 °C for 1 min. Negative and positive controls were added for results verification. The results were considered positive for all sigmoidal curve and cycle thresholds (Ct) < 38 curves following manufacture instruction.

### Statistical data management and analysis

Laboratory results of detected pathogens as well as the additional clinical and radiological characteristics were compared using Chi-square test to examine the relationship between two qualitative variables, while Fisher’s Exact test was used to examine the relationship between two qualitative variables when the expected count is less than 5 in more than 20% of cells. Student T Test was used to assess the statistical significance of the difference between two study group means. All statistical analyses and calculations were performed using the SPSS 27.0 statistical software package. Evaluations and comments were made at a significant level of p-value lower than 0.05.

## Results

Demographics, clinical characteristics and patient co-morbidities collected are listed in Table 1. The study included 313 (52.3%) Females and 286 males (47.7%). Out of these, 134 (22.4%) patients were > 15 years, 341 (56.9%) patients between 15 and 60 years and 124 (20.7%) patients were more than 60 years. The mean age of the study population was 36.29 years (SE = 0.98), with a median age of 35.10 years and an interquartile range of 36.16 years. The skewness value was 0.046. Most of the patients (58.2%) had symptoms of fever, cough, dyspnea, diarrhea, runny nose, and sore throat. Comorbidities conditions were detected in 46.4% (278/599) of patients, which included hypertension (30.9%), diabetes (26.9%), chronic cardiac disease (26.9%), and Asthma (21.5%).

**Fig. 1:**
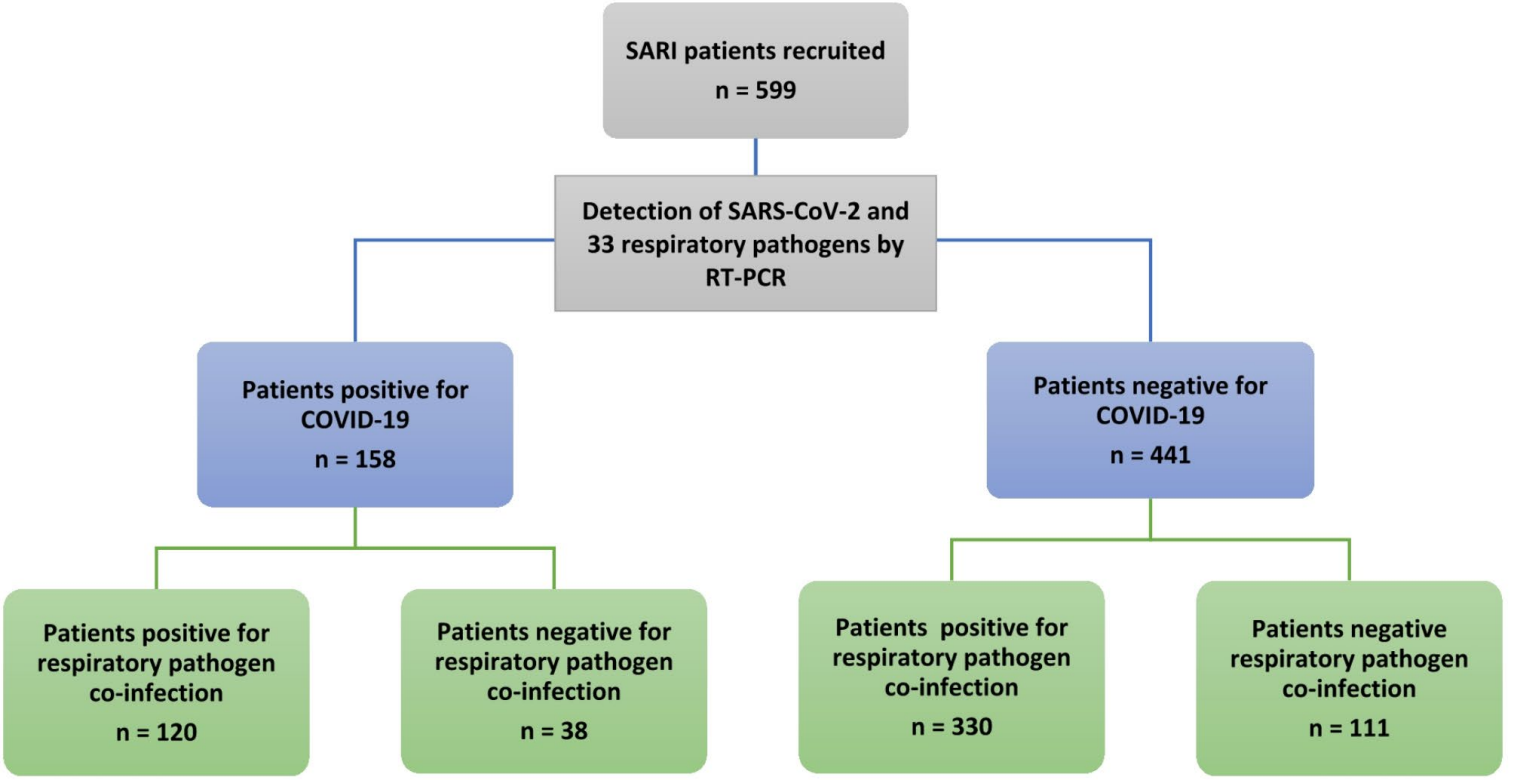
Flowchart for study participants.

Out of the 599 tested samples, 158 samples showed positive SARS-CoV-2 results. All samples were evaluated for other respiratory pathogens in which 31 pathogens were identified in positive and negative COVID-19 patients, including 12 bacteria, 18 viruses, and 1 fungus with a detection rate of 75% (450/599) (Fig. 1) (Table 2). Bacterial co-infections rate was 39%, in which *K. pneumoniae* was the most common (5.3%), while viral co-infection rate was 61%, in which HCOV HKU1 (6.2%) was the most common. Solitary infections were detected in the patient samples, 38 (6.3%) with SARS-CoV-2, 41 (6.8%) with other viruses, and 53 (8.9%) with bacteria.

**Table 1:** Demographics and clinical characteristics of enrolled patients.

	PCR COVID-19		*P* Value
	Positive	Negative	
	n (%)	n (%)	
Gender			
Male	79 (27.6%)	207 (72.4%)	0.509
Female	79 (25.2%)	234 (74.8%)	
Age Groups			
0–15 Years	9 (6.7%)	125 (93.3%)	0.000*
15–40 Years	49 (24.6%)	150 (75.4%)	
40–60 Years	52 (36.6%)	90 (63.4%)	
Above 60 Years	48 (36.7%)	76 (61.3%)	
Symptoms			
Fever	99 (28.4%)	250 (71.6%)	0.192
Cough	126 (25.9%)	361 (74.1%)	0.559
Dyspnea	102 (25.7%)	295 (74.3%)	0.594
Diarrhea	50 (27.6%)	131 (72.4%)	0.649
Loss of smell	51 (31.3%)	112 (68.7%)	0.095
Loss of taste	48 (31.4%)	105 (68.6%)	0.104
Runny nose	59 (21.7%)	213 (78.3%)	0.018*
Sore throat	68 (26.2%)	192 (73.8%)	0.913
Comorbidity	90 (32.4%)	188 (67.6%)	0.002*
Vascular event	19 (22.1%)	67 (77.9%)	0.330
Neurological event	11 (18.3%)	49 (81.7%)	0.136
Hypertension	35 (40.7%)	51 (59.3%)	0.001*
Diabetes	29 (38.7%)	46 (61.3%)	0.010*
Chronic cardiac disease	18 (24%)	57 (76%)	0.617
Asthma	15 (25%)	45 (75%)	0.799
Chronic respiratory disease	11 (23.4%)	36 (76.6%)	0.630
Obesity	8 (32%)	17 (68%)	0.515
Chronic liver disease	7 (41.2%)	10 (58.8%)	0.16
Chronic neurological disease	7 (50%)	7 (50%)	0.042*
Chronic renal disease	3 25%)	9 (75%)	0.913
Chronic hematological disorder	3 (30%)	7 (70%)	0.793
Pregnant	1 (11.1%)	8 (88.9%)	0.295
Seasonal Influenza vaccination in last 6 months	2 (25%)	6 (75%)	0.929
Immunodeficiency	2 (33.3%)	4 (66.7%)	0.698

Values in the same row and subtable not sharing the same subscript are significantly different at *p* < .05 in the two-sided test of equality for column proportions.

**Table 2:** Respiratory pathogens detected in the studied samples.

Pathogen	*n* (%)
Positive for any infection	488 (81.5)
Pathogens detected in the study sample	
*SARS-CoV-2*	158 (26.4)
*Other corona viruses*	175 (29.2)
*Para-Influenza viruses*	118 (19.7)
*Influenza viruses*	111 (19.5)
*Adenoviruses*	70(11.7)
*Enteroviruses*	64 (10.7)
*Human Rhinovirus*	50 (8.3)
*Respiratory Syncytial virus (RSV)*	41 (6.8)
*Bocavirus*	28 (4.7)
Other viruses	
*Mycoplasma Pneumoniae*	36 (6.0)
*Pneumocystis Jirovecii*	67 (11.2)
Chlamydia pneumoniae	32 (5.3)
*Gram positive organisms*	89 (14.9)
*Gram negative organisms*	249 (41.6)
Co-infections in the study sample	
Single infection	132 (22)
Double	92 (15.4)
Triple or more	233 (44.1)

Out of 158 COVID-19 patients, 120 were found to be co-infected with other respiratory pathogens, in which 5.5% were infected with single pathogen, 4.9% were co-infected with two pathogens, and 14.1% were co-infected with three pathogens and more (Table 3). In Negative COVID-19 patients, 330 patients were found to be co-infected with other respiratory pathogens, in which 20.1% were infected with single pathogen, 13.5% were co-infected with two pathogens, 8.4% were co-infected with three pathogens and 25.6% were co-infected with more than three pathogens (Table 4).

Analysis of respiratory pathogens co-infection with age group, comorbidities and symptoms are listed in Table 5. A chi square group difference between number of co-infections among patients with or without COVID-19 and age groups indicated a significant association of moderate effect (χ2 (21, *n* = 450) = 97.98 *p* = .001, Cramer’s V = 0.259), while with comorbidities indicated a significant association (χ2 (7, *n* = 450) = 15.03 *p* = .036 for hypertension and = 16.79 *p* = .019 for diabetes; and insignificant association (χ2 (7, *n* = 488) = 11.81 *p* = .107 for chronic cardiac diseases.

**Fig. 2:**
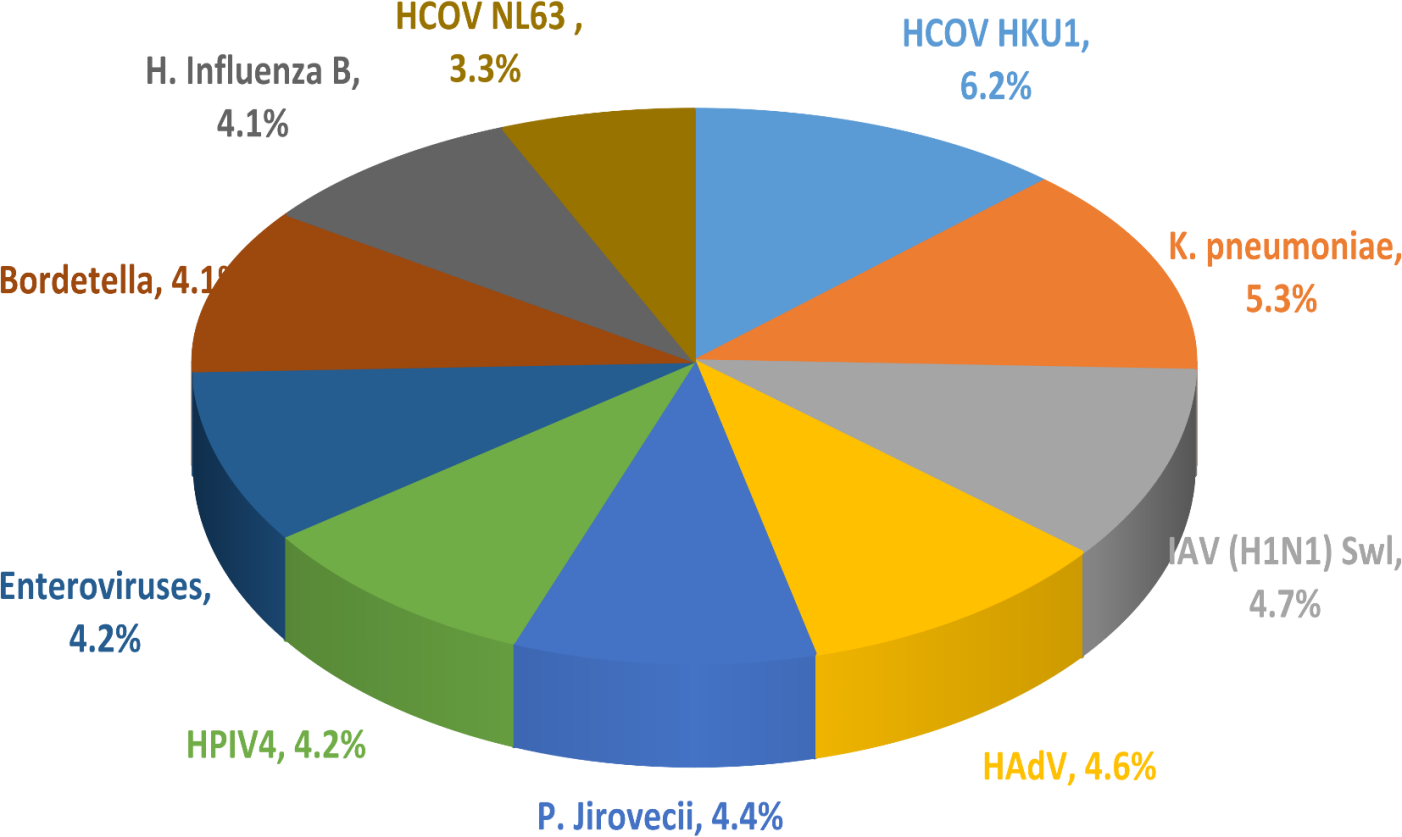
Most dominant respiratory pathogens found in all patients.

The most dominant pathogens based on the calculated cumulative score “total infections present in more than 60% of total number of patients” found in all patients are 6.2% HCOV HKU1, 5.3% *K. pneumoniae*, 4.7% IAV (H1N1) Swl, 4.6% HAdV, 4.4% *P. Jirovecii*, 4.2% HPIV4, 4.2% EV, 4.1% Bordetella, 4.1% *H. Influenza* B and 3.3% HCOV NL63 (Fig. 2).

**Table 3:** Respiratory pathogens co-infections detected in COVID-19 patients.

No. of infection				
	Double* (Coinfection)	3* Coinfections	> 3 Coinfections	Total*
	n (%)	n (%)	n (%)	n (%)
HCOV HKU1	1(3.7)	4(16.7)	18(26.1)	23(19.2)
*K. pneumoniae*	13(48.1)	3(12.5)	9(13)	25(20.8)
IAV (H1N1) Swl	0(0)	2(8.3)	10(14.5)	12(10)
HAdV	2(7.4)	4(16.7)	14(20.3)	20(16.7)
*P. Jirovecii*	1(3.7)	2(8.3)	21(30.4)	24(20)
HPIV4	0(0)	2(8.3)	18(26.1)	20(16.7)
EV	0(0)	2(8.3)	20(29)	22(18.3)
Bordetella	1(3.7)	4(16.7)	10(14.5)	15(12.5)
H. Influenza B	0(0)	0(0)	0(0)	0(0)
HCOV NL63	0(0)	1(4.2)	12(17.4)	13(10.8)
S. aureus	2(7.4)	0(0)	5(7.2)	7(5.8)
HRV	0(0)	0(0)	14(20.3)	14(11. 7)
IBV	0(0)	1(4.2)	(0)	1(0.8)
M. Catarrhalis	1(3.7)	3(12.5)	7(10.1)	11(9.2)
HPIV1	0(0)	4(16.7)	14(20.3)	18(15)
*S. pneumoniae*	2(7.4)	1(4.2)	5(7.2)	8(6.7)
Salmonella	0(0)	0(0)	12(17.4)	12(10)
HRSVA and HRSVB	0(0)	2(8.3)	12(17.4)	14(11.7)
HCOV OC43	0(0)	1(4.2)	10(14.5)	11(9.17)
KLePSa L. pneumophila/ L. longbeache	0(0)	1(4.2)	11(15.9)	12(10)
HCOV 229E	0(0)	1(4.2)	9(13)	10(8.3)
*M. pneumoniae*	0(0)	4(16.7)	5(7.2)	9(7.5)
HBoV	0(0)	0(0)	8(11.6)	8(6. 7)
*C. pneumoniae*	1(3.7)	1(4.2)	6(8.7)	8(6. 7)
HPIV2	0(0)	1(4.2)	7(10.1)	8(6. 7)
IAV	1(3.7)	1(4.2)	2(2.9)	4(3. 33)
ICV	0(0)	0(0)	2(2.9)	2(1. 7)
HPeV	1(3.7)	1(4.2)	3(4.3)	4(3.3)
H. influenza	0(0)	0(0)	1(1.4)	1(0.8)
HPIV3	1(3.7)	1(4.2)	4(5.8)	2(1.7)
HMPVA and B	0(0)	1(4.2)	0(0)	1(0.8)

*Double infections: Patients positive for covid19 and one respiratory pathogen.

*Triple infections: patients positive for covid19 and two respiratory pathogens.

*Total: the percentage calculated according to the total number of patients who have coinfections with covid19 (232).

**Table 4:** Respiratory pathogens co-infections detected in negative COVID-19 patients.

	No. of infection				
	Single* infection	Double (Coinfection)	3 Coinfections	> 3 Coinfections	Total*
	*n* (%)	*n* (%)	*n* (%)	*n* (%)	*n* (%)
HCOV HKU1	5(5.3)	7(10.6)	13(31.7)	47(37.6)	72(21.9)
*K. pneumoniae*	19(23.5)	14(21.2)	7(17.1)	16(12.8)	56(17)
IAV (H1N1) Swl	1(1.4)	7(10.6)	7(17.1)	45(36)	60(18.2)
HAdV	8(11.4)	12(18.2)	3(7.3)	27(21.6)	50(15.2)
*P. Jirovecii*	0(0)	2(3)	6(14.6)	35(28)	43(13)
HPIV4	3(4.7)	6(9.1)	5(12.2)	30(24)	44(13.3)
EV	3(4.7)	10(15.2)	3(7.3)	26(20.8)	42(12.7)
Bordetella	6(9.5)	8(12.1)	10(24.4)	24(19.2)	48(14.5)
H. Influenza B	1(1.6)	7(10.6)	5(12.2)	0(0)	13(4)
HCOV NL63	3(6)	2(3)	10(24.4)	22(17.6)	37(11.2)
S. aureus	13(26)	3(4.5)	1(2.4)	26(20.8)	43(13)
HRV	10(20)	7(10.6)	0(0)	19(15.2)	36(10.9)
IBV	2(4.1)	3(4.5)	7(17.1)	0(0)	12(3.6)
M. Catarrhalis	3(6.25)	3(4.5)	2(4.9)	29(23.2)	37(11.2)
HPIV1	0(0)	5(7.6)	3(7.3)	20(16)	28(8.59)
*S. pneumoniae*	6(13.6)	3(4.5)	0(0)	27(21.6)	36(10.9)
Salmonella	0(0)	3(4.5)	1(2.4)	27(21.6)	31(9.4)
HRSVA and HRSVB	2(4.87)	1(1.5)	4(9.8)	20(16)	27(8.2)
HCOV OC43	1(2.6)	3(4.5)	5(12.2)	18(14.4)	27(8.2)
KLePSa L. pneumophila/ L. longbeache	1(2.6)	2(3)	4(9.8)	19(15.2	26(7.9)
HCOV 229E	3(8.3)	4(6.1)	6(14.6)	13(10.4)	26(7.9)
*M. pneumoniae*	0(0)	2(3)	4(9.8)	21(16.8)	27(8.2)
HBoV	0(0)	4(6.1)	3(7.38)	13(10.4)	20(6.1)
*C. pneumoniae*	2(8.7)	3(4.5)	3(7.38)	7(5.6)	15(4.5)
HPIV2	0(0)	0(0)	1(2.48)	13(10.4)	14(4.2)
IAV	1(5)	1(1.5)	1(2.48)	13(10.4)	16(4.8)
ICV	1(5.6)	3(4.5)	2(4.9)	10(8)	16(4.8)
HPeV	1(7.1)	2(3)	2(4.9)	4(3.2)	9(2.7)
H. influenza	2(15.4)	3(4.5)	2(4.9)	5(4)	12(3.6)
HPIV3	0(0)	0(0)	1(2.4)	5(4)	6(1.8)
HMPVA and B	1(12.5)	2(3)	2(4.9)	2(1.6)	7(2.1)

*Double infections: Two pathogens detected.

*Triple infections: Three pathogens detected.

*More than triple infections: More than three pathogens detected.

*Total: The percentage calculated according to the total number of patients who have coinfections negative covid19.

**Table 5:** Comparison between COVID-19 positive and negative patients with co-infections.

	POS COVID − 19 with coinfection (*n* = 120)	NEG COVID-19 with infections (*n* = 330)	*P*-value
Gender			0.853
Male	57 (47.5)	160 (48.5)	
Female	63 (52.5)	170 (51.5)	
Age group (years)			< 0.001
Less than 15 years	8 (6.7)	108 (32.7)	
15-	29 (24.2)	91 (27.6)	
40-	42 (35)	68 (20.6)	
≥ 60	41 (34.2)	63 (19.1)	
Comorbidities			
Hypertension	29 (24.2)	45 (13.6)	0.008
Diabetes	24 (20)	39 (11.8)	0.027
Chronic cardiac disease	15 (12.5)	54 (16.4)	0.314
Chronic respiratory disease	10 ( 8.3)	34 (10.3)	0.534
Obesity	6 (5)	15 (4.5)	0.84
Symptoms			
Fever	81 (67.5)	217 (65.8)	0.730
Cough	100 (83.3)	275 (83.3)	1
Dyspnea	81 (67.5)	230 (69.7)	0.656
Diarrhea	35 (29.2)	103 (31.2)	0.677
Loss of smell	34 (28.3)	69 (20.9)	0.097
Loss of taste	34 (28.3)	62 (18.8)	0.029
Running nose	40 (33.3)	149 (45.2)	0.025
Sore throat	51 (42.5)	131 (39.7)	0.592
Vascular event	10 (8.3)	35 (10.6)	0.477
Severity			
Mild	42 (19.8)	170 (51.5)	0.008
Moderate	52 (32.3)	109 (33)	
Severe	26 (33.8)	51 (15.5)	
Healthcare type			
Home isolation	29 (24)	80 (24.2)	0.022
Hospital intermediate care	15 (12.5)	25 (7.5%)	
Hospital ward	45 (37.5)	165 (50)	
ICU admission	20 (16.6)	27 (8.2)	
Mortality	14 (11.7)	12 (3.6)	0.001
Laboratory tests (Median (IQR))			
TLC	7.62 (3.71)	8 (4.48)	0.921
Absolute neutrophilic count	4.84 (3.52)	4.8 (3.37)	0.226
Absolute lymphocytic count	1.31 (2.1)	1.9 ( 2.2)	0.037
Neutr/Lymph ratio	3.3 (4.78)	2.42 (2.95)	0.005
Absolute monocytic count	0.61 (0.6)	0.6 (0.57)	0.698
Platelet	223 (104)	275 (150)	< 0.001
Ferritin	1.14 (2.47)	0.98 (1.59)	0.127
D- dimer	23 (19)	19 (14)	0.022
ALT	23 (19)	20 (17)	0.068
CRP	375 (578)	217 (290)	0.003

## Discussion

Severe acute respiratory infection (SARI), the third leading cause of death globally, represent a challenge for healthcare systems, especially in low- and middle-income countries^3,4^. A number of respiratory viruses and bacterial etiologies in patients that met SARI criteria have been reported^21,22^. During COVID-19 pandemic, we experienced a surge of acute respiratory infections caused by several pathogens, including SARS-CoV-2, influenza, RSV, and other seasonal virus and bacteria. The co-circulation of these pathogens increases pressure on healthcare systems, with outbreaks of respiratory infection leading to increased primary care consultations and hospitalizations, particularly for patients with comorbidities and risk factors for severe outcomes^18^.

After the first confirmed case of COVID-19 in Egypt in February 2020, SARS-CoV-2 were widely spread with increasing in the number of cases as reported in the official website of the Egyptian Ministry of health (https://www.care.gov.eg/EgyptCare/index.aspx)^23^. Co-infection with bacteria, fungi, and respiratory viruses in SARS-CoV-2 is of particular importance due to the possibility of increased morbidity and mortality^21^. Therefore, physicians need to be cognizant about excluding other treatable respiratory pathogens^22^. The rate of co-infection among these pathogens is unknown. Therefore, a positive diagnostic test for another infection does not exclude the need for other micro-organism testing^21^.

Our results revealed that out of 599 patients presented with SARI from October 2020 till June 2022, 158 samples showed positive SARS-CoV-2 results. Our positivity rate is within the range found in recent studies reported during same time period during COVID-19 pandemic; 23.1% positive for SARS-CoV-2 among Indian patients with SARI/influenza like illness from April to September 2020^24^; 52% positive for SARS-CoV-2 among Irish SARI cases from July 2021 and April 2022^25^; from 10% to 24% in the EU/EEA depending on the week of report during 2023^12^; and 89% among adult SARI patient in United States between March 2020 and April 2023^26^. This disparity in the prevalence of detection of SARS-CoV-2 during this period may be due to regional variability^12^, the difference in the inclusion criteria of the cases in the different studies, such as the highest trend reported from U.S. may be attributed to age of included patients (median age was 60 years), meanwhile, our study included age group < 15 - >60 years old. Young age group showed significant low SARS-CoV-2 infection, which matches with studies that reported the frequency of positivity on RT-PCR for SARS-CoV-2 was higher in adults than in adolescence and both were higher than in children^27^. As reported by Goldstein et al. that children aged under 10y have significantly lower susceptibility to SARS-CoV-2 infection, while adults aged over 60y have elevated susceptibility to infection^28^. Our results showed that the middle-aged and the elderly made up higher percentage of all patients. Similarly, different studies indicated that patients infected with SARS-CoV-2 are mostly middle-aged and elderly^15,29^.

In the current study, all patients presenting as SARI, either positive or negative PCR COVID-19, had the same common symptoms including fever, cough, sore throat, dyspnea, which matches with other report of same time period^30^; loss of taste (ageusia) preceding the onset of respiratory symptoms has also been reported to be with SARS-CoV-2 infected patients^11^, and was significantly linked to SARS-CoV-2 infected group in our study; running nose was significantly linked to SARI patients with SARS-CoV-2 negative test, which in concordance with that reported by Khutade et al.^7^. Diabetes and hypertension were significantly associated with COVID-19 infection, which matches with that reported by Akhtar et al.^31^.

After selection of all factors in univariate analysis with a p-value ≤ 0.35, multivariate analysis with different approaches was used to select the most significant factors that influence in-hospital mortality in studies conducted on COVID-19 Egyptian patients during the same period. Being elderly (> 60 years), having a shorter duration of complaint, having a high NLR, and having a higher CT-SS (> 20) were all significant independent predictors of mortality (*P* < .05 for all), meanwhile, ischemic heart disease, hypertension, hemodialysis and stroke were non- significant^32^.

Our results showed that COVID-19 patients with certain comorbidities (hypertension, and diabetes) had a significant incidence of coinfection (p-value 0.008 and 0.027 respectively). This matches with Trifonova et al.^33^, who reported that in co-infected adults with SARS-CoV-2 and other respiratory viruses, the proportion of patients with concomitant diseases was 70%, and the most common were hypertension (48.9%) and diabetes (24.5%). Mukherjee et al.^34^ found a high prevalence of comorbid conditions among hospitalized patients with laboratory confirmed viral SARI, including 50% with hypertension, 34% with diabetes mellitus, 17% with CKD and 15% with coronary artery disease.

All samples were evaluated for 33 respiratory pathogens in which 31 pathogens were identified (12 bacteria, 18 viruses, and 1 fungus) with a detection rate of positive for any infection 81.5% (488/599), and positive for co-infection 75% (450/599). Zhang et al., reported positive rate of respiratory pathogen strains of 40.18%, and 4.69% of mixed infection were detected during same period of COVID-19 pandemic among patients with respiratory tract infection, whereas the patients were screened for only 13 pathogen^3^; 72.5% reported by Hanchi et al. from January 2018 to December 2019 among SARIs children for at least one respiratory pathogen and 23.3% of them were co-infected with more than one pathogen^35^, and 35.5% tested positive for only SARS-CoV-2 and influenza, including 0.8% co-infection^34^.This disparity in the prevalence of detection of respiratory pathogens may be due to the difference in the inclusion criteria of the cases in the different studies, the population, age groups, the geographical location, the seasons, and the panel used for the molecular diagnosis^7,21^. The high rate of positivity in our study can be explained by the use of larger panel of respiratory pathogens as well as more sensitive molecular techniques, which detect 33 respiratory pathogens simultaneously.

In our study, 120 positive COVID-19 patients, and 330 negative COVID-19 patients were co-infected with other respiratory pathogens. The results suggest that co-infections rates are significantly higher among SARS-CoV-2 negative SARI group as compared to positive group. This matches with Sapra et al., finding and this may be due to viral interference and competitive advantage of SARS-CoV-2 in modulating the host immunity^24^ .

For positive co-infection studied samples, bacterial co-infections rate was 39%, in which *K. pneumoniae* is the most common (5.3%), while viral co-infections rate was 61% in which HCOV HKU (6.2%) was the most common and this is in concordance with other studies that reported HCOV HKU1 as the most dominant virus^15,24^. These results were contradicting with other research findings in which bacterial pathogens were the dominant co-infections^25,36^. Similar studies stated that the most common co-pathogens among COVID-19 patients were HCOV HKU1^15^, *Klebsiella* species (spp.)^36^, *K. pneumoniae*^29,37^, in addition to other pathogens like *P. jirovecii*^29^, and EV^29,38^.

The high rate of fungal co-infection representing *P. jirovecii* in the studied patients, could be explained by the immunodeficiency brought on by SARS-CoV-2 infection, which may make room for the growth of this opportunistic fungus as reported by Dueñas D et al.^29^. Co-infection with *P. jirovecii* and SARS-CoV-2 has been reported in a patient with progressive hypoxemic respiratory failure and CD41 lymphocytopenia^39^. Therefore, physicians may consider additional diagnostic testing such as serum-beta-D-glucan for *P. jirovecii*, especially when there are other characteristics supporting co-infections and classical risk factors for *P. pneumonia*^26^.

Regarding the profile of the detected respiratory pathogens in the present study, the most common pathogens were SARS-COV2 (158/26.4%), other corona viruses (175/29.2%), para-influenza viruses (118/19.7%), influenza viruses (111/19.7%), adeno-viruses (70/11.7%), gram positive organism (249/41.6%), gram negative organism (89 /14.9%), *p. jirovecii* (67/11.2%), and *mycoplasma pneumoniae* (36/6%). Nevertheless, human rhinovirus and RSV were only detected in 50/8.3% and 41/6.8% of cases, respectively. These results are comparable to list of pathogen reported by WHO for differential diagnosis of SARI^23^. Our study was conducted during COVID pandemic, this makes SARS-CoV-2 at the top of the profile^23^. A unique wide range of pathogens were screened in our study that make our results more informative about Egyptian patients than, Fahim et al.^40^, who studied co-infection of only SARS-COV2 and Influenza viruses among acute respiratory infections between 2020 and 2022. Their results showed that 35.5% were positive for viruses, including 0.8% co-infection. Of them, 69.2% were co-infected with Flu A/H, 17.3% Flu-B, and 13.5% Flu A/H1^3^. In recent studies, it was noticeable that Human rhinovirus (HRV) was the most detected respiratory pathogen^37,38,41,42^. However, this is in contrary to this current profile and may be explained by COVID pandemic throughout the study period.

In SARS-CoV-2 patients specific bacterial co-infecting pathogens were identified, with *K. pneumoniae* the most common (20.8%). A finding which has previously been reported by Sreenath et al.^26^ who support the systemic use of antibiotics in patients with severe SARS-CoV-2 *pneumoniae* based on the higher proportion of patients with co-infections in his cohort result, with rapid de-escalation based on respiratory PCR/culture results^22^.This finding may reflect high rates of antimicrobial use for admitted COVID-19 patients to treat documented or presumed bacterial co-infections. Thus, it is important to study the occurrence, type, and intended antimicrobial agent use in SARS-COV-2 patients in order to develop additional strategies for the optimal use of antimicrobial agents in this population^21^.

Co-infections among SARS-COV2 infected group were significantly associate with severity of COVID-19 infection, ICU admission, and higher mortality. Same significant association was raised by Fahim et al.^40^ and Sharma et al.^30^. This could be explained by the hypothesis that viruses elevate bacterial co-infection by impairing the host’s immune response, disrupting epithelial barrier integrity, expression of surface receptors and adhesion proteins, direct binding of virus to bacteria, altering nutritional immunity, and affecting the bacterial biofilm. Similarly, the bacteria enhance viral infection by altering the host’s immune response, up-regulation of adhesion proteins, and activation of viral proteins^43^.

Interestingly, our study demonstrated that SARS-CoV-2 co-infected with influenza A (4/3.33%) and C viruses (2/1.7%), and very low trend co-infected with influenza B virus (1/0.8%), although the latter was found to be a co-infecting virus with all other respiratory viruses tested (12/3.6%). This finding was reported by studies investigating simultaneous circulation of SARS-CoV-2 and influenza for fear of putting an additional burden on health care systems in 2020, 2021 and the first half of 2022. They confirmed a low percentage of patients co-infected with both viruses 2.45% by Boussarsar et al.^44^ and 0.7% by Dao et al.^45^. Both studies confirmed more co-infections in critically ill patients compared to mono-infected patients.

Solitary infection was detected in 22% among our studied samples, in which 38 (6.3%) were infected with SARS-CoV-2, 41 (6.8%) with other viruses, and 53 (8.9%) with bacteria. The high proportion of co-infections suggests that testing for SARS-CoV-2 and other common respiratory pathogens should be carried out to ensure accurate diagnoses, prompt patient treatment, and appropriate isolation^46^.

Our finding of high trend of co-infections, underlines the usefulness of multiplex PCR in the etiological diagnosis of respiratory infections due to their high sensitivity, specificity, and rapidity^42^, especially when it is difficult to identify the etiologic pathogen on the basis of symptoms only. Indeed, respiratory infections are characterized by low specificity of clinical assessment^46^. A study conducted by Lee et al.^47^, showed that the adoption of a molecular respiratory panel with a short turnaround time provides significant benefit and improves decision-making for patient management by reducing antibiotic use and chest X-ray prescription, and finally decreasing the length of inpatients stay. Furthermore, Brendish et al.^48^ report that the rapid turnaround time was strongly correlated with earlier hospital discharge and early antibiotic discontinuation. They suggest that there is an early “window of opportunity” for the results of the molecular diagnostic tests to change patient management and support the turnaround time as a crucial determinant of medical decision-making. However, the cost-effectiveness of a molecular diagnosis for respiratory pathogens in pediatric patients remains to be determined. Despite the fact that the diagnostic strategy for the respiratory pathogens co-infection in SARI patients based on multiplex PCR is costly, it is likely reduce the overall cost of care as it significantly reduces the length of hospital stay, antibiotic use, better case management and the preparedness for future pandemics^48^ .

## Data Availability

All data generated or analyzed during this study are included in this published article.
